# Identification of KHDC1L, a DUX4-regulated protein, as a novel plasma biomarker in facioscapulohumeral muscular dystrophy

**DOI:** 10.1093/hmg/ddaf183

**Published:** 2025-12-12

**Authors:** Nicholas A Sutliff, Emily Chao, Sean R Bennett, Yee Nip, Omar Lakhdari, David A Canton, Yiming Zhu, Stephen J Tapscott

**Affiliations:** Fred Hutchinson Cancer Center, Human Biology Division, 1100 Fairview Ave N, Seattle, WA 98109, USA; Avidity Biosciences, 3020 Callan Rd, San Diego, CA 92121, USA; Fred Hutchinson Cancer Center, Human Biology Division, 1100 Fairview Ave N, Seattle, WA 98109, USA; Fred Hutchinson Cancer Center, Human Biology Division, 1100 Fairview Ave N, Seattle, WA 98109, USA; Avidity Biosciences, 3020 Callan Rd, San Diego, CA 92121, USA; Avidity Biosciences, 3020 Callan Rd, San Diego, CA 92121, USA; Avidity Biosciences, 3020 Callan Rd, San Diego, CA 92121, USA; Fred Hutchinson Cancer Center, Human Biology Division, 1100 Fairview Ave N, Seattle, WA 98109, USA

**Keywords:** facioscapulohumeral dystrophy, KHDC1L, DUX4, circulating biomarker, plasma

## Abstract

Facioscapulohumeral muscular dystrophy (FSHD) is caused by aberrant expression of the double homeobox transcription factor DUX4 in skeletal muscle. Because direct measurement of DUX4 in FSHD muscle is technically challenging, DUX4-regulated transcripts in muscle biopsies have been used as surrogates; however, this approach is invasive, limited to a single muscle, and less suitable for repeated monitoring. Thus, we sought to identify DUX4-regulated circulating biomarkers that could integrate DUX4 activity across all affected muscles and enable more frequent measurement. We performed mass spectrometry on conditioned media from DUX4-inducible immortalized human myoblasts (MB135iDUX4) and identified a top candidate—KHDC1L, the protein product of a DUX4-regulated mRNA previously shown to correlate with DUX4 expression in muscle. Western blotting confirmed KHDC1L release into the supernatant of DUX4-expressing cells. Plasma profiling demonstrated elevated KHDC1L levels in individuals with FSHD compared to healthy controls, supporting its role as a circulating readout of DUX4 activity. These findings suggest that plasma KHDC1L is a potential pharmacodynamic marker of DUX4 activity, providing a minimally invasive tool for disease monitoring and a potential response marker to evaluate emerging FSHD therapies.

## Introduction

Facioscapulohumeral dystrophy (FSHD) is the third most common inherited muscular dystrophy, affecting ~ 1 in 8000–1:20000 individuals [1]. Individuals with FSHD typically experience progressive weakness of facial, scapular, and humeral muscles, with later involvement of trunk and lower limbs [2]. The molecular cause of FSHD is aberrant expression of the double homeobox transcription factor DUX4 in skeletal muscle [3, 4]. DUX4 is normally expressed in early embryogenesis, where it regulates zygotic genome activation [5], and is epigenetically silenced in somatic tissues, including skeletal muscle. In FSHD skeletal muscle, aberrant expression of DUX4 re-activates the early embryonic program and results in progressive muscle dysfunction and damage [6–8]. Several studies have shown that in muscle biopsies the RNA abundance of genes regulated by DUX4 correlate with disease activity in each muscle [8–11]. But biopsies are invasive, reflect activity in only a single muscle, and are less suitable for repeated measurements of dynamic changes.

Several studies have explored circulating biomarkers in FSHD. Analysis of gene expression in peripheral blood cells did not identify differential expression of DUX4 target genes nor the expression of PAX7-regulated genes in FSHD compared to control circulating cells [12]; however, a subsequent study using a refined set of PAX7-regulated genes showed a correlation with FSHD, particularly in older subjects [13]. Other studies have shown increased expression of some miRNAs [14, 15], the inflammatory proteins IL-6 and TNF [16, 17], and complement factors [18] in the circulation of FSHD compared to control subjects; however the lack of specificity for FSHD (e.g. more direct link to DUX4) and marginal discrimination have limited their clinical utility. In addition to circulating biomarkers, quantitative MRI of whole-body muscle correlates with disease activity and is under evaluation as a progression endpoint [19–23]. Automated segmentation can provide muscle-specific measures of volume, fat fraction and oedema [24], yet detectable radiological changes often emerge only after months and necessitate sizeable cohorts.

There remains a critical need for a biomarker that directly reflects whole-body DUX4 activity and can be sampled repeatedly over time in a minimally invasive manner. In this study, we identify KHDC1L, a gene regulated by DUX4 with negligible expression in adult somatic tissues, as a secreted protein detectable in FSHD plasma. We show that plasma KHDC1L levels are elevated in FSHD subjects compared to unaffected controls, establishing circulating KHDC1L as a promising candidate biomarker. By providing a direct and systemic readout of DUX4 activity, circulating KHDC1L has the potential to accelerate therapeutic development in FSHD.

## Results

### Identification of KHDC1L as a candidate secreted protein induced by DUX4

To capture proteins released from DUX4-expressing myoblasts, we profiled the conditioned medium of MB135 cells engineered with doxycycline-inducible DUX4 (MB135iDUX4). This cell line has previously been reported to exhibit robust induction of DUX4 and its downstream transcriptomic targets, consistent with the expression pattern observed for wild-type DUX4 in FSHD muscle tissue [25]. MB135iDUX4 cell supernatant was collected after continuous doxycycline treatment to induce DUX4 in regular growth medium conditions followed by additional time in serum-free media without additives except for doxycycline. Label-free quantitative mass spectrometry of concentrated supernatant from two biological replicates yielded sixteen proteins that were ≥ 2-fold enriched over uninduced controls and predicted to be secreted (Table 1).

**Table 1:** Candidate secreted proteins in conditioned media.

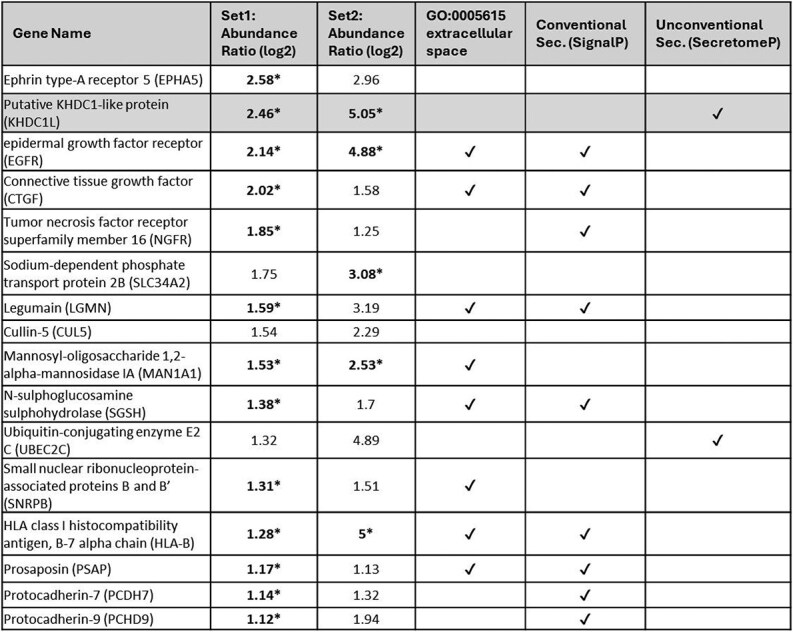

**Figure 1:**
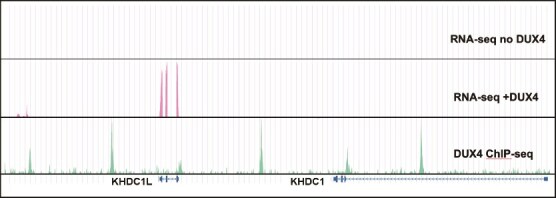
DUX4 induces expression of KHDC1L and has a ChIP-seq peak in the promoter region. Presentation of prior RNA sequencing (GSE85461) and DUX4 ChIP-seq (GSE33838) data showing the KHDC1L and KHDC1 gene regions. MB135 cells do not have detectable expression of DUX4 (RNA-seq no DUX4) and the expression of DUX4 robustly induces KHDC1L RNA (RNA-seq + DUX4). KHDC1 is not expressed in MB135 cells either with or without DUX4. A DUX4 ChIP-seq peak is present at the promoter region of KHDC1L, indicating DUX4 binding at that region.

**Figure 2:**
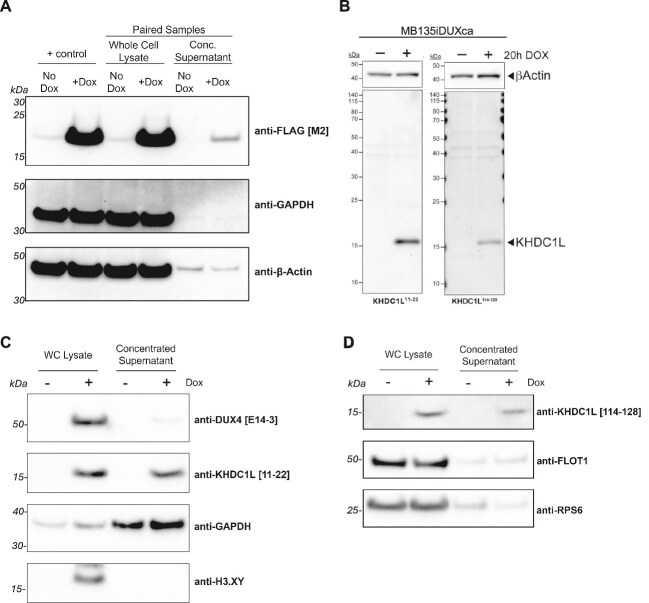
(A) KHDC1L is released from MB135 myoblasts. MB135 cells transduced with a doxycycline-inducible 3xFLAG-tagged KHDC1L showed release of the tagged KHDC1L protein in the concentrated supernatant following doxycycline induction. Actin was present with or without DUX4 induction and is associated with exosomes but we did not determine whether the actin signal represented exosomes. (B) DUX4 induction of KHDC1L mRNA correlates with detection of KHDC1L protein by the KHDC1L 11–22 and the KHDC1L 114–128 monoclonal antibodies. Panels show immunoblot detection of KHDC1L protein in cells treated with doxycycline (+ DOX) to induce DUX4 and KHDC1L mRNA expression. Left panel: Antibody to KHDC1L peptide 11–22; right panel, antibody to KHDC1L peptide 114–128. (C, D) KHDC1L protein is present in the supernatant of cells expressing DUX4 but not in the same cells without DUX4 expression. (C) Doxycycline treatment (+) has been shown to induce KHDC1L mRNA and the KHDC1L 11–22 monoclonal antibody detects the KHDC1L protein in both the whole cell lysate proteins (WC lysate) and in the concentrated supernatant from the tissue culture media. GAPDH is a secreted protein during micronutrient starvation and physiological stress [28, 29] and serves as a positive control for supernatant proteins and H3.XY and DUX4 are nuclear proteins that serve as controls showing that intracellular proteins are not in the supernatant. (D) Similar KHDC1L induction in WC lysate and concentrated supernatant was obtained with KHDC1L 114–128 monoclonal antibody. FLOT1 is an exosome associated protein showing low abundance in the supernatant; RPS6 is primarily localized in the cytoplasm.

Detection of SLC34A2, a previously reported DUX4-target [26], validated the inducible cell line model and screening methodology. KHDC1L was prioritized because (i) It is robustly induced by DUX4 and chromatin immunoprecipitation (ChIP) shows a promoter proximal DUX4 binding site (Fig. 1 and Supplemental Fig. 1) [25]; (ii) Its mRNA strongly discriminates FSHD from control muscle biopsies [10, 11, 25, 27]; (iii) It lacks appreciable expression in adult somatic tissues based on the Human Protein Atlas; and (iv) SecretomeP 2.0 annotated it as being an unconventional secreted protein. Because DUX4 expression triggers apoptosis within 24–48 h, we asked whether KHDC1L release simply reflected loss of membrane integrity. Parental MB135 cells were engineered to express 3 × FLAG-KHDC1L under doxycycline control and confirmed to not show significant changes in proliferation or viability via an MTS assay (Supplemental Fig. 2). Upon induction, these cells secreted 3xFLAG-KHDC1L into the medium within 20 h despite the absence of DUX4, supportive of an active export mechanism (Fig. 2A).

### Development of reagents to detect KHDC1L

To facilitate the development of orthogonal assays to measure KHDC1L, we generated antibodies against the target (Supplemental Fig. 3). Two mouse monoclonal antibodies recognizing non-overlapping unique KHDC1L epitopes (amino acids 11–22 and 114–128) were generated. Both antibodies detected a single ~ 17 kDa band by immunoblot following DUX4 induction, near the predicted size of the endogenous KHDC1L protein (Fig. 2B). Immunoblotting of MB135iDUX4 culture supernatants with clone 11–22 (Fig. 2C) and clone 114–128 (Fig. 2D) confirmed that KHDC1L was released from cells after DUX4 induction. Nuclear markers (DUX4, H3.X/Y) were absent, while GAPDH served as a secretion-control protein (Fig. 2C). The difference in GAPDH abundance between experiments likely reflects serum-free versus standard growth media used in the FLAG-KHDC1L experiments. FLOT1 is an exosome associated protein showing low abundance in the supernatant; RPS6 is primarily localized in the cytoplasm (Fig. 2D).

**Figure 3:**
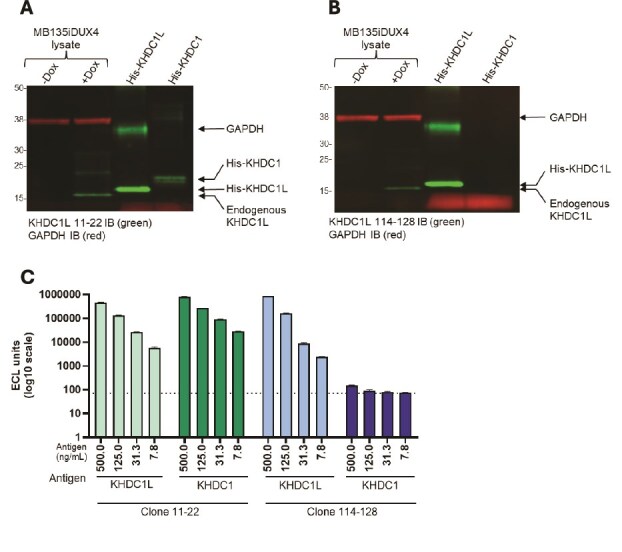
(A, B) specificity of anti-KHDC1L clones 11–22 and 114–128 assessed using immunoblots against KHDC1, a related KHDC1L family member with high sequence homology. Similar to Fig. 2, both mAbs detect KHDC1L upon DUX4 induction in MB135iDUX4 cell lysate (lanes 1 and 2, both blots; 20ug total cell lysate). (A) When tested against the two recombinant family members, clone 11–22 detected both recombinant His-tagged KHDC1L and His-tagged KHDC1. (B) Clone 114–128 is selective for recombinant His-tagged KHDC1L. Recombinant proteins were loaded on the gel at 62.5 ng for His-KHDC1L and 70.2 ng for His-KHDC1. (C) Specificity of anti-KHDC1L clones 11–22 and 114–128 evaluated by ECLIA. Recombinant His-tagged KHDC1 and KHDC1L were directly coated to plates at concentrations listed, followed by incubation with either anti-KHDC1L clone 11–22 or 114–128 (1ug/mL). Detection was with anti-mouse antibodies conjugated to SULFO tag (1ug/mL). Dotted line denotes background level.

The binding affinity and specificity of both antibodies were evaluated to characterize their interaction profiles. The affinity for each antibody and recombinant KHDC1L was determined to be < 50 pM for clone 114–128 and 2.08 nM for clone 11–22, as measured by bilayer interferometry (BLI; Supplemental Table 1). Cross-reactivity of the antibodies to KHDC1, a closely related family member with high sequence homology, was assessed by Western blot (Fig. 3A and B) and a plate-based direct detection assay (Fig. 3C). In both assays, clone 11–22 failed to differentiate between the two family members; however, clone 114–128 was found to be specific for KHDC1L.

### Detection of KHDC1L in FSHD plasma

**Figure 4:**
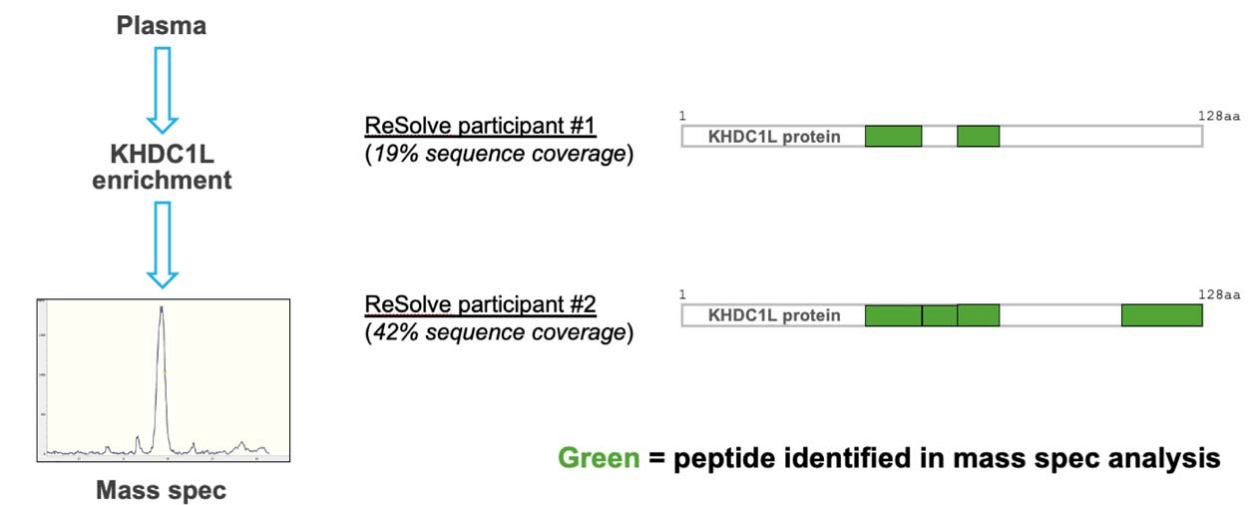
Mass spectrometry detection of KHDC1L-specific peptides in FSHD patient plasma samples. Enrichment from plasma was performed using a biotinylated aptamer targeting KHDC1L coupled to Dynamag beads. Eluted proteins were digested with trypsin and analyzed using a PRM-PASEF targeted acquisition on a timsTOF HT mass spectrometer (Bruker).

Three different assay formats were evaluated to detect KHDC1L in FSHD plasma, starting with mass spectrometry following target enrichment. Pulldowns were performed with a biotinylated KHDC1L-specific aptamer coupled to streptavidin magnetic beads. Eluted proteins were digested with trypsin and analyzed using a PRM-PASEF targeted acquisition on a timsTOF HT mass spectrometer (Fig. 4). Two unique KHDC1L peptides were detected in FSHD patient plasma #1 and four unique peptides in patient plasma #2, yielding sequence coverage of 19% and 42%, respectively. No KHDC1L-specific peptides were identified following enrichment from one healthy volunteer plasma, potentially representing lower plasma expression in healthy volunteers compared to disease (Supplemental Table 2). While this methodology confirmed the detection of KHDC1L in FSHD plasma, the assay lacked reproducibility and was not further developed.

**Figure 5:**
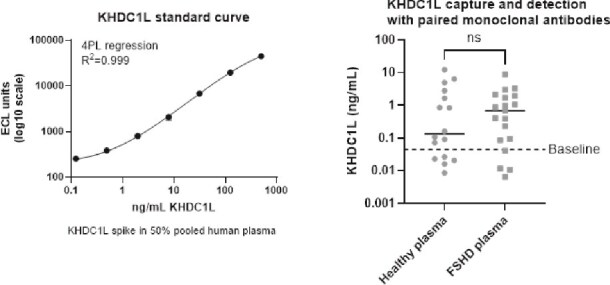
ECLIA detection of circulating KHDC1L levels in FSHD patient plasma compared to healthy volunteer plasma. (left) standard curve for the ECLIA assay generated using recombinant KHDC1L spiked in 50% human plasma to concentrations of 500, 125, 31.25, 7.81, 1.95, 0.49, 0.12 and 0 ng/ml. The standard curve was interpolated to allow quantitation of endogenous KHDC1L levels in plasma. (right) analysis of the levels of plasma KHDC1L in plasma samples from FSHD patients (n = 20; samples from Resolve natural history study) versus unaffected controls (n = 20; BioIVT).

We next evaluated a sandwich electrochemiluminescence immunoassay (ECLIA) to detect KHDC1L in FSHD plasma. The ECLIA assay using KHDC1L-specific clone 114–128 as capture and clone 11–22 as a detector demonstrated a lower limit of quantitation of ~ 0.5 ng/ml and < 1% cross-reactivity to the family member KHDC1. Plasma obtained from FSHD participants in the ReSolve Natural History study and healthy volunteers was analyzed using this assay (Fig. 5). Median levels of plasma KHDC1L were trending higher in FSHD plasma but failed to reach significance (healthy volunteers median = 0.1349 ng/ml, n = 16; FSHD patients median = 0.6986 ng/ml, n = 19; *P* = 0.6588).

**Figure 6:**
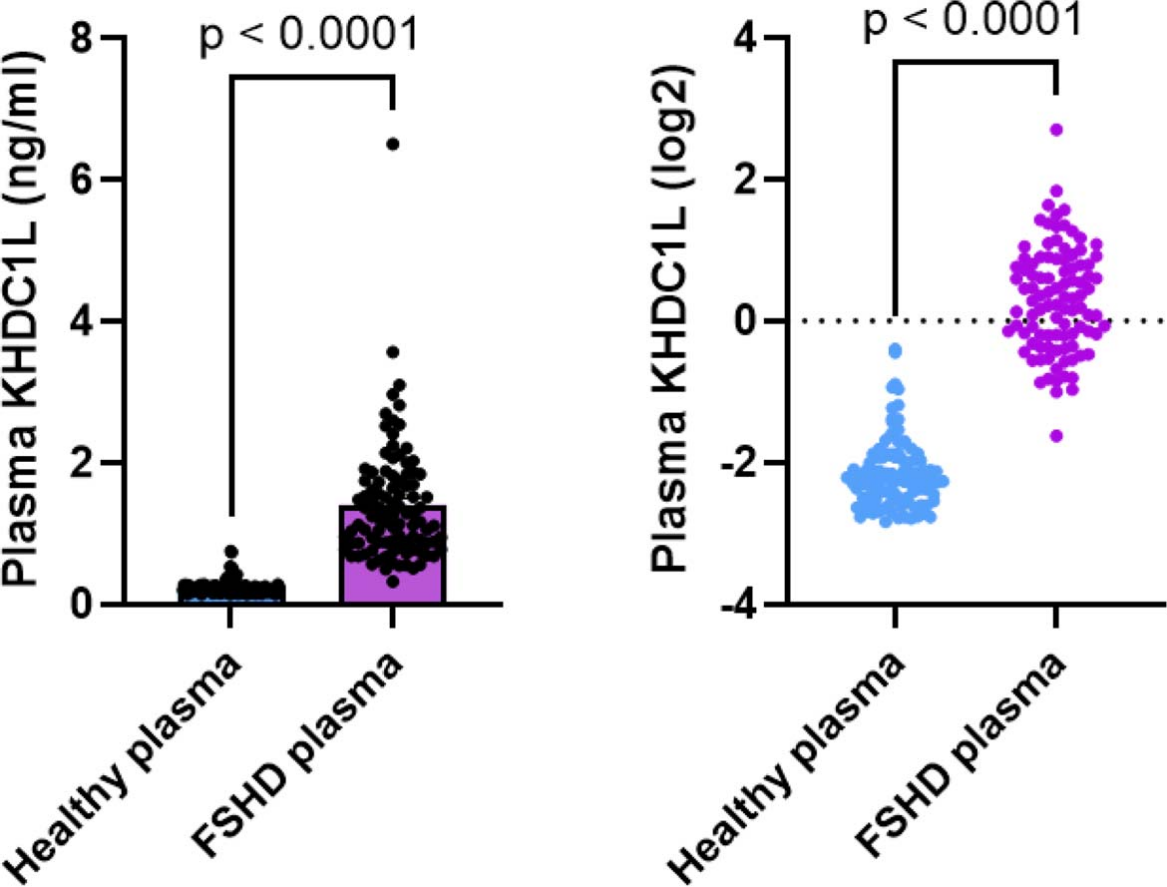
Aptamer-mediated detection of KHDC1L levels in plasma. The SomaScan 7K platform (SomaLogic, Boulder CO) was used to quantitate levels of KHDC1L in plasma samples from subjects with FSHD (n = 98; samples from ReSolve natural history study) versus age- and sex-matched healthy volunteers (n = 100; BioIVT). Levels of KHDC1L in plasma were interpolated from a standard curve of recombinant KHDC1L. Circulating levels in FSHD plasma were significantly higher than healthy volunteers (^****^P < 0.0001).

Finally, we employed the SomaScan 7K aptamer proteomics platform, which uses specific DNA aptamers designed to bind plasma proteins in a multiplexed format. Plasma from individuals with FSHD and healthy volunteers were analyzed and KHDC1L levels were interpolated from a standard curve (Fig. 6). Circulating levels of KHDC1L were significantly elevated in FSHD plasma compared to age- and sex-matched healthy volunteers (FSHD plasma mean = 1.408 ng/ml, n = 98; healthy volunteers mean = 0.2412 ng/ml, n = 100; ^****^P < 0.0001).

Taken together, discovery proteomics, cell-based secretion assays, three independent detection platforms, and patient plasma analyses converge to identify KHDC1L as a DUX4-regulated secreted biomarker candidate that is elevated in FSHD, justifying continued research (e.g. longitudinal and pharmacodynamic studies) to further evaluate circulating KHDC1L in FSHD.

## Discussion

In this study, we used mass spectrometry to identify KHDC1L as a DUX4-regulated protein that is released from myoblasts and significantly elevated in the plasma of individuals with FSHD. KHDC1L is a well-characterized DUX4 target gene [25], previously identified as a dysregulated transcript in FSHD muscle biopsies [10, 11, 25, 27], and also expressed in other DUX4 contexts such as zygotic gene activation (ZGA) and certain cancers [5, 30]. Our data provide the first evidence that KHDC1L is not only upregulated at the transcript level, but is also detectable in FSHD plasma, distinguishing FSHD from control plasma samples.

Despite its role as a downstream target gene of DUX4, the biological function of KHDC1L remains poorly understood. Expression of mouse KHDC1A (a family member) in human HeLa cells showed that it localized to the endoplasmic reticulum, induced apoptosis, and inhibited RNA translation [31]. In humans, KHDC1L is located on chromosome 6q13 and shares ~ 84% identity with its family member KHDC1. The Human Protein Atlas database indicates KHDC1L expression mostly in testis and some areas of the brain, with low but detectable levels in thymus. Expression of KHDC1L in a cancer cell line promoted cell proliferation and inhibited apoptosis [32]. Additional research is needed to fully characterize the biological function and impact of KHDC1L expression in FSHD and to understand the mechanisms governing its secretion, stability, and tissue specificity in vivo.

One limitation is that our study relied on cross-sectional analysis of FSHD plasma while longitudinal sampling will be required to understand intra-individual variability, temporal stability and how KHDC1L levels track with disease progression and therapeutic intervention. A highly sensitive and reproducible assay will be critical for detecting DUX4-regulated factors like KHDC1L, which are expected to be at very low levels in plasma given that only a small fraction of muscle fibers express DUX4 at any given time.

Despite these caveats, plasma KHDC1L shows potential as a surrogate readout of DUX4 activity, which differentiates it from the other reported circulating biomarkers that measure downstream pathology including muscle damage and inflammation. Because DUX4 expression is variable both between and within FSHD skeletal muscles, a blood-based biomarker offers a ‘global average’ of disease burden. However, technical challenges remain. Differences in KHDC1L signal across platforms—including mass spectrometry, SomaScan, and ECLIA—highlight the need for assay optimization, particularly regarding detection thresholds, antibody/aptamer binding affinities, and potential matrix effects. The use of paired ECLIA antibodies with varying affinities, as shown in Supplemental Table 1, may also contribute to inter-assay variability.

Notably, ongoing trials of DUX4-targeting therapies have begun incorporating KHDC1L as an endpoint, measuring reductions in its protein level as a pharmacodynamic readout (clinicaltrials.gov NCT05747924), using the assay described in this study. Early interim data are encouraging: treatment with a DUX4-targeting siRNA led to a measurable drop (~40%) in plasma KHDC1L levels within just a few months. Together, our findings provide the first evidence to support plasma KHDC1L as a candidate DUX4-regulated biomarker in FSHD that can be used to monitor disease progression and therapeutic response.

## Material and methods

### RNA-sequencing and ChIP-sequencing analysis

RNA sequencing data were obtained from the publicly available dataset GSE85461 to assess the transcriptional response of KHDC1L and KHDC1 to DUX4 expression in MB135iDUX4 cells. Cells either lacking DUX4 expression (‘RNA-seq no DUX4’) or transduced to express DUX4 (‘RNA-seq + DUX4’) were analyzed. Expression levels of KHDC1L and KHDC1 were visualized at the gene level using genome browser tracks. Chromatin immunoprecipitation followed by high-throughput sequencing (ChIP-seq) data for DUX4 were obtained from dataset GSE33838 to identify genomic binding sites of DUX4. The promoter regions of KHDC1L and KHDC1 were specifically examined for enrichment of DUX4 binding. ChIP-seq peaks were visualized and aligned with the RNA-seq tracks to assess potential regulatory interactions.

### Generation of supernatant for mass spectrometry analysis

15 cm plates of near confluent MB135iDUX4 cells [33] were treated with 1 ug/ml doxycycline, or not, in regular growth culture conditions (F-10 media with 20% FBS, 1% Pen-Strep, 10 ng/ml bFGF, and 1 uM dexamethasone), then washed three times in phosphate buffered saline (PBS) to remove serum proteins followed by additional time in additive-free F10 media plus 1 ug/ml doxycycline for ‘+ Dox’ samples. Sample set 1 was cultured for 6 h in regular culture conditions and then 6 h in additive-free F-10 media. Sample set 2 was cultured for 12 h in regular growth conditions and 4 h in additive-free F-10 media. For each sample set, two parallel plates were treated with doxycycline for biological duplicates, and two plates were similarly processed without the doxycycline treatment for control biological replicates without DUX4 expression. For sample harvest, 30 ml of the supernatant was centrifuged at 3260 g for 10 min at 4°C to remove floating cells and debris. The supernatant was concentrated in two steps: first with an Amicon Ultra-15 3 kDa MW cut-off filter unit centrifuged in a swinging bucket rotor at 4000 g to decrease the volume to ~ 200–500 ul, and second with an Amicon Ultra-0.5 3 kDa MW cutoff filter centrifuged 15 min at 14000 g at 4°C to a final volume of ~ 50 ul. NuPage LDS Sample Buffer and NuPage Reducing Agent were added to 1x and then the concentrated samples were run ~ 1 cm into a NuPage 4%–12% Bis-Tris precast gel at 100 V with NuPage MOPS SDS Running Buffer. The gel was stained with GelCode Blue and then the entire ~ 1 cm lane containing protein for each sample was excised with a clean razor blade, as well as an additional ~ 1 cm lane containing no sample as a blank control. The gel slices were placed into clean 1.5 ml tubes and then submitted to the Fred Hutchinson Cancer Center Proteomics Core for additional pre-processing (trypsin digestion, and dehydration) followed by label-free mass spectroscopy identification of proteins in each of the four samples: two doxycycline treated and two untreated.

### Mass spectrometry data analysis

Mass spectrometry data was analyzed in Proteome Discoverer for label-free quantification of relative protein abundance (log2 fold-change). Statistical significance was determined by the abundance ratio adjusted p-values calculated in Proteome Discoverer.

### Western blot on cell lysates

For Western blots (Fig. 2B), cell lysates were prepared in RIPA buffer with protease and phosphatase inhibitors and 10 ug of protein was mixed with an equal volume of 2X Laemmli sample buffer and run on a NuPage 12% Bis-Tris precast gel in NuPage MES SDS Running Buffer. Proteins were transferred to a 0.2 um PVDF membrane in NuPage Transfer Buffer with 20% methanol for 50 min at 30 V in an XCell II Blot Module. Membranes were blocked in phosphate buffered saline containing 0.1% Tween-20 and 5% nonfat dry milk before overnight incubation at 4 degrees C with anti-FLAG M2 (1:1000; MilliporeSigma), primary monoclonal antibody using a 1:500 dilution of a 1.63 mg/ml (anti-KHDC1L 11–12) or a 1.71 mg/ml (anti-KHDC1L 114–128) stock. For Western blots in Fig. 3A and B, 20 ug whole cell lysate and 62.5 ng recombinant His-KHDC1L protein (Novus Biologics) and 70.2 ng recombinant His-KHDC1 (Origene) were run per lane. Samples were run on 4%–12% NuPAGE gels, semi-dry transfer to PVDF, blocked in 5% milk, then detected with LiCor secondary antibodies.

### Binding affinity of mouse monoclonal KHDC1L antibodies by biolayer interference (BLI)

Clones 114–128 and 11–22 binding affinities were determined to KHDC1L-AviTag by biolayer interferometry (BLI, Gator Bio). Briefly, antibodies (4 ug/ml) were immobilized onto anti-Mouse IgG Fc biosensor tips and exposed to a dilution range of biotinylated KHDC1L-AviTag from 100 to 3.125 nM. Similarly, KHDC1L-AviTag was immobilized onto streptavidin biosensor tips and exposed to a dilution range of antibodies from 50 to 1.5625 nM. Dissociation constants (KD) were determined.

### Proliferation of inducible 3xFLAG-KHDC1L cells

Cell proliferation was measured using the CellTiter 96 AQueous One Solution Cell Proliferation Assay, according to manufacturer’s recommendations. Briefly, MB135 cells engineered with inducible 3xFLAG-KHDC1L were plated in triplicate at 500 cells/well in a 96-well plate and treated with and without doxycycline. Cell viability/proliferation was evaluated once a day for 4 days by addition of 20 ul One Solution reagent, incubation for 2 h at 37°C, 5% CO_2_, followed by measurement of formazan product at 490 nm. Data is shown as background corrected absorbance.

### Western blot on tissue culture supernatant

For Western blots shown in Fig. 2C and D, culture supernatant (25 ml) from each of two 15-cm plates of near confluent MB135iDUX4 cells treated, or not, with 1 ug/ml doxycycline for four hours then replaced with serum-free media. The harvested supernatant was supplemented with protease/phosphatase inhibitors and then centrifuged and concentrated to a final volume of 50–100 ul as described above for the preparation of the samples for mass spectroscopy. Whole cell lysates were prepared in RIPA buffer with protease/phosphatase inhibitors from the same cells and quantified by BCA (bicinchoninic acid) assay. 10 ug protein for whole cell lysates and 33% volume of concentrated supernatant had NuPage LDS Sample Buffer added to 1X and beta-mercaptoethanol to 5%. Protein samples were run at 100 V on a 4%–12% Bis-Tris NuPage pre-cast gel with NuPage MES SDS Running Buffer and then transferred to a 0.2um PVDF membrane in NuPage Transfer Buffer with 10% methanol at 30 V for 1 h in an X-Cell Surelock Mini-Cell electrophoresis and blot system. Membranes were blocked in blocking buffer (TBS with 0.05% Tween20 and 5% nonfat dry milk) for 1 h before overnight incubation with primary antibodies diluted in blocking buffer at 4°C. Then the membranes were washed 3 times with wash buffer (TBS with 0.05% Tween20) for 15 min each, incubated with HRP-conjugated secondary antibodies diluted in blocking buffer for 1 h, and washed 3 times with TBS with 0.05% Tween20 for 15 min each before developing with ECL and imaging with a BioRad ChemiDoc. For the Western blot in Fig. 2, MB135i3xFLAG-KHDC1L were harvested with the same protocol but the cells were maintained in serum-containing growth media. The primary antibodies used were anti-DUX4 [E14–3] rabbit mAb (1:1000), anti-KHDC1L mouse mAb [11–22] (1:1000), anti-GAPDH [6C5] mouse mAb (1:5000), anti-H3.XY rat antisera (1:10), anti-KHDC1L mouse mAb [114–128], anti-FLOT1 [D2V7J] rabbit mAb (1:1000), and anti-RPS6 rabbit mAb [5G10] (1:1000).

### Enrichment and mass spectrometry detection

Per manufacturer’s instructions, SOMAmer Reagent 21 804–15 was diluted to 50 nM in SB buffer (40 mM HEPES pH 7.5, 100 mM NaCl, 5 mM MgCl_2_, 5 mM KCl, 1 mM EDTA, 20 mM NaOH, 0.05% Tween-20, pH 7.5), heated to 95°C for 10 min, then slowly cooled to 37°C at a rate of 0.1°C per second. The SOMAmer was coupled to streptavidin magnetic beads (Phenomenex), resuspended in a total volume of 100uL SB buffer and kept on ice protected from light. Pulldowns were performed for 2 hours on plasma from (1) healthy volunteer and (2) FSHD patients (250 uL each). Beads were washed three times by rotating in cold HSE buffer (20 mM HEPES pH 7.2, 150 mM NaCl, 1% Triton X-100), with magnetic capture and removal of buffer after each wash. Captured proteins were eluted in 20 ul denaturing buffer and heating for 5 min at 95°C. Supernatant was collected, reduced and alkylated, and then diluted to 100 ul in buffer containing trypsin for overnight digestion. Digested peptides (20 ul) were loaded onto an Evotip (Evosep 1 system) and injected onto column for analysis. Data was acquired using a PRM-PASEF targeted acquisition on a timsTOF HT (Bruker) and analyzed in Skyline. Resulting peptide peaks were manually inspected with a false-discovery rate of ≤ 1%.

### ECLIA assay

Anti-KHDC1L clone 114–128 was coated on high binding 96-well plates at 1 ug/ml and blocked with MSD buffer A. Standards were prepared in 50% plasma, 500, 125, 31.25, 7.81. 1.95, 0.49 and 0.12 ng/ml. Duplicate 50 ul patient samples and standards were bound to the plate for 2.5 h. Detection was with SULFO-tagged anti-KHDC1L clone 11–22 on a MESO Sector S 600MM.

### MSD assay

Recombinant His-tagged KHDC1L was purchased from Novus Biologics and His-tagged KHDC1 was purchased from Origene. Both proteins were coated on high-binding 96-well plates at 500, 125, 31.3 and 7.8 ng/ml overnight in phosphate buffered saline. Anti-KHDC1L mAbs 11–22 and 114–128 were added at 1 ug/ml and incubated for 1 h. Detection was performed with 1 ug/ml SULFO-tagged anti-mouse antibodies labeled using MSD GOLD Sulfo-Tag NHS-Ester (cat. # R91AO-1). Plates were read on MESO Sector S 600MM.

### Analysis of KHDC1L in patient plasma

Plasma was obtained from people living with FSHD (n = 98, Resolve Natural History study) and age- and sex-matched healthy volunteers (n = 100, BioIVT). All samples were collected with IRB approval and consent forms. Plasma aliquots (55 ul) were shipped to Somalogic (Boulder CO) and analyzed with the SomaScan 7K platform per manufacturers standard operating procedures. Levels of plasma KHDC1L were interpolated from a standard curve of recombinant KHDC1L diluted in healthy volunteer plasma and are reported as concentration in ng/mL and log2 concentration for each patient sample.

## Supplementary Material

Sutliff-Suppl-Figs_ddaf183

Sutliff-Suppl-Tables_ddaf183

## Notes

*Conflict of interest statement:* S.J.T. has consulting and advisory positions with companies developing therapeutics for FSHD, including the Advisory Board for Renogenyx, and a Consultant Agreement and Sponsored Research Agreement with Avidity Biosciences. E.C., O.L, D.A.C, and Y.Z. are employees of Avidity Biosciences. The Fred Hutchinson Cancer Center and Avidity Biosciences have filed a patent application on materials related to this manuscript. The other authors declare no conflict of interest.
