# Supplementary material for: Identification of KHDC1L, a DUX4-regulated protein, as a novel plasma biomarker in facioscapulohumeral muscular dystrophy
Source: Hum Mol Genet. 2025 Dec 12;35(2):ddaf183. doi: 10.1093/hmg/ddaf183 (PMC13158228; doi:10.1093/hmg/ddaf183)
Supplement: Sutliff-Suppl-Figs_ddaf183 [file sutliff-suppl-figs_ddaf183.pdf]

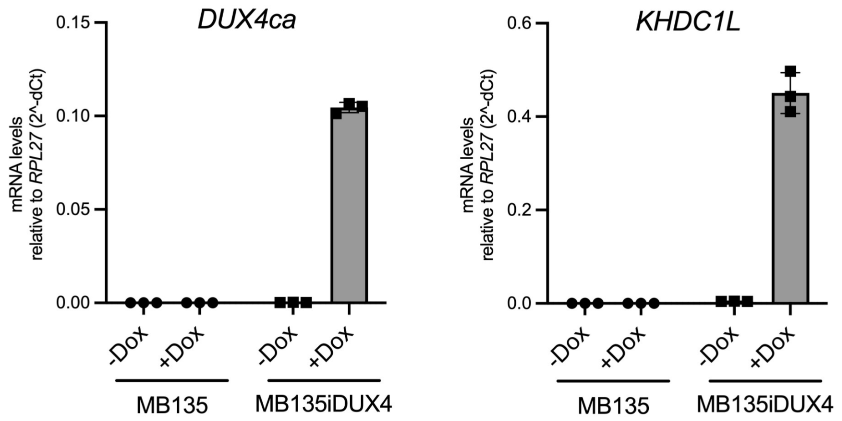

Supplemental Figure 1: KHDC1L mRNA is not induced by doxycycline in the parental MB135iDUX4 cells, where doxycycline induces the codon altered DUX4 (DUX4ca) mRNA, but not in the parental MB135 cells that do not express DUX4ca. Cells were grown without (-Dox) or with (+Dox) 1 ug/mL doxycycline continuously for 20 hr followed by RT-qPCR analysis of DUX4ca and KHDC1L expression. Data are shown as relative expression normalized to RPL27 (mean ± SD of biological replicates, n=3).

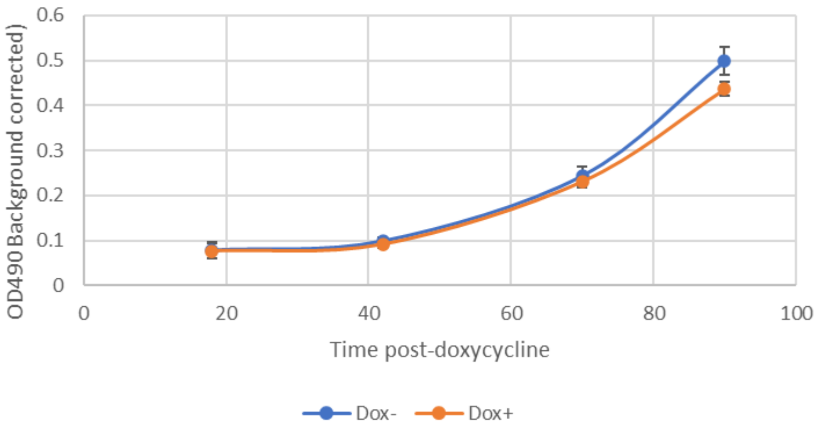

Supplemental Figure 2: Proliferation/viability of inducible FLAG-KHDC1L expression in MB135 cells. MB135 cells engineered with inducible FLAG-KHDC1L were plated at 500 cells/well in a 96-well plate and treated with and without doxycycline. Proliferation was evaluated once a day for 4 days via reduction of MTS tetrazolium measured at 490nm (MTS assay).

Peptides used for immunization:

11-22 amino acids

114-128 amino acids

Alignment between KHDC1L and family member KHDC1

```
KHDC1L  1     ------------------------------------------------------------  0

KHDC1   1     MLSAFQRLFRVLFVIETVSEYGVLIFIYGWPFLQTLAMLLIGTVSFHLWIRRNRERNSRS  60

                                   11-22aa
KHDC1L  1     -------------MAVGTSALSKEPWWTLPENFHSPMVFHMEEDQEELIFG-LDDTYLRC  46
                           |::|||||||:||||||:|||#||||||||||||||||#:#||||||
KHDC1   61    GKTRCRSKRSEQSMDMGTSALSKKPWWTLPQNFHAPMVFHMEEDQEELIFGHG-DTYLRC  119


KHDC1L  47    IELHSHTLIQLERCFTATGQTRVTVVGPPMAKQWLLLMFHCVGSQDSKCHARGLKMLERV  106
              ||:|||||||||:#||||||||||||||::|:||||:||:|||||||::|||||#|||||
KHDC1   120   IEVHSHTLIQLESWFTATGQTRVTVVGPHRARQWLLHMFCCVGSQDSYHHARGLEMLERV  179

                     114-128aa
KHDC1L  107   RSQPLTNDDLVTSVSLPPYTGD------------------------------------  128
              |||||||||||||#|:||||||
KHDC1   180   RSQPLTNDDLVTSISVPPYTGDLSLAPRISGTVCLSVPQPSPYQVIGCSGFHLSSLYP  237
```

Supplemental Figure 3: Sequence alignment between KHDC1L and the related family member, KHDC1, with the two immunization peptides highlighted by red boxes.
