# Supplementary material for: Identification of KHDC1L, a DUX4-regulated protein, as a novel plasma biomarker in facioscapulohumeral muscular dystrophy
Source: Hum Mol Genet. 2025 Dec 12;35(2):ddaf183. doi: 10.1093/hmg/ddaf183 (PMC13158228; doi:10.1093/hmg/ddaf183)
Supplement: Sutliff-Suppl-Tables_ddaf183 [file sutliff-suppl-tables_ddaf183.pdf]

Supplemental Table 1: Binding affinities of mouse anti-KHDC1L clones to biotinylated KHDC1L-AviTag by biolayer interference (BLI)

Assay #	Immobilized to tip	Sample	KD (nM)	koff (1/s)	kon (1/Ms)	Full R^2	Full X^2
1	Clone 114-128	KHDC1L-AviTag	OR	OR	7.81E+04	0.9841	3.1806
2	KHDC1L-AviTag	114-128	2.40	1.33E-03	5.54E+05	0.9878	1.9664
3	Clone 11-22	KHDC1L-AviTag	2.08	2.35E-04	1.13E+05	0.9734	3.2683
4	KHDC1L-AviTag	11-22	OR	OR	3.45E+04	0.9674	0.6997

OR = Out of Range of Detection (i.e., < 50 pM)

Supplemental Table 2: KHDC1L peptides detected by mass spectrometry

Plasma sample	KHDC1L peptides identified	Protein coverage (%)
Healthy volunteer	No peptides detected	0%
FSHD plasma #1	C[Carbamidomethyl]IELHSHTLIQLER	19%
	VTVVGPPMAK	
FSHD plasma #2	C[Carbamidomethyl]IELHSHTLIQLER	42%
	C[Carbamidomethyl]FTATGQTR	
	VTVVGPPMAK	
	SQPLTNDDLVTSVSLPPYTGD	

Supplemental Table 3: Members of the ReSolve Network

Institution	Affiliation
**University of Kansas Medical Center:** Jeffrey Statland MD, Site PI; Mazen Dimachkie MD, Sub-Investigator; Constantine Farmakidis MD, Sub-Investigator, Mamantha Pasnoor MD, Sub-Investigator, Duaa Jabari MD, Sub-Investigator; Omar Jawdat MD, Sub-Investigator; Melissa Currence PTA, Clinical Evaluator; Sandhya Sasidharan DPT, Clinical Evaluator; Kiley Higgs BS CCRP, Research Project Manager; Michaela Walker MPH CCRP, Research Project Manager; Rebecca Clay BS CCRP, Clinical Research Coordinator	Department of Neurology, Kansas City, KS, United States of America
**University of Rochester Medical Center:** Rabi Tawil MD, Site PI; Johanna Hamel MD, Sub-Investigator; Kate Eichinger DPT, Clinical Evaluator; Leann Lewis MSc CCRP, Clinical Research Coordinator	Department of Neurology, Rochester, NY, United States of America
**The Ohio State University:** Samantha LoRusso MD, Site PI; Bakri Elsheikh MD, Site PI; W. David Arnold MD, Sub-Investigator; Kristina Kelly DPT PhD, Clinical Evaluator; Marco Tellez NCMA CCRP, Clinical Research Coordinator	Department of Neurology, Columbus, OH, United States of America
**University of Washington:** Leo H. Wang MD PhD, Site PI; Matthew K Preston MD, Sub-Investigator; Catherine Kieu DPT, Clinical Evaluator; Aliya Shabbir Clinical Research Coordinator	Department of Neurology, Seattle, WA, United States of America
**University of Utah:** Russell Butterfield MD PhD, Site PI; Clark Moser MD, Sub-Investigator; Melissa McIntyre DPT PhD Candidate, Clinical Evaluator; Amelia Wilson DPT, Clinical Evaluator; Sarah Moldt MBA CCRC PMP, Clinical Research Coordinator	Department of Neurology, Department of Pediatrics, Department of Physical Therapy & Athletic Training, Salt Lake City, UT, United States of America
**Kennedy Krieger Institute:** Katheryn Wagner MD PhD, Site PI; Doris Leung MD PhD, Site PI; Andrea Jaworek DPT, Clinical Evaluator; Nikia Stinson DPT, Clinical Evaluator; Mary Yep BS, Clinical Research Coordinator	Center for Genetic Muscle Disorders, Department of Neurology at the Johns Hopkins University School of Medicine, Baltimore, MD, United States of America
**University of California Los Angeles:** Perry Shieh MD PhD, Site PI; Christy Skura DPT, Clinical Evaluator; Jennifer Huynh BS Clinical Research Coordinator	Department of Neurology at the David Geffen School of Medicine, Los Angeles, CA, United States of America
**Virginia Commonwealth University:** Nicholas Johnson MD, Site PI; Amanda Butler DPT, Clinical Evaluator; Aileen Jones DPT, Clinical Coordinator; Jodie Howell LPN CCRP, Clinical Research Coordinator	Center for Inherited Muscle Research, Department of Neurology, Richmond, VA, United States of America
**Radboud University Medical Center:** Karlien Mul MD PhD, Site PI	Department of Neurology, Donders Institute for Brain, Cognition and Behavior, Radboud University Medical Center, Nijmegen, The Netherlands
**NeuroMuscular Omnicentre Clinical Center Milano:** Valeria Sansone MD, Site PI; Elena Carraro MD, Site PI; Valentina Franchino PT, Clinical Evaluator; Erica DiNatale, Clinical Research Coordinator; Michael Nani, Clinical Research Coordinator	Department of Neurology, Milan, Italy
**Chu de Nice:** Sabrina Sacconi MD PhD, Site PI; Luisa Villa MD, Sub-Investigator; Giulia Tammam MD, Sub-Investigator; Jeremy Garcia PT, Clinical Evaluator; Manuela Gambella, Clinical Research Coordinator; Sandray Ayari PhD, Clinical Research Coordinator	Peripheral Nervous System and Muscle Department, Rare Neuromuscular Diseases Reference Centre, Competence Centre for Neurogenetic Diseases, Institut of Research on Cancer and Aging of Nice, Department of Neurology, Nice, France
